# Snow Surface Microbial Diversity at the Detection Limit within the Vicinity of the Concordia Station, Antarctica

**DOI:** 10.3390/life13010113

**Published:** 2022-12-30

**Authors:** Alessandro Napoli, Claudia Coleine, Nikea J. Ulrich, Ralf Moeller, Daniela Billi, Laura Selbmann

**Affiliations:** 1 Department of Biology, University of Rome Tor Vergata, 00133 Rome, Italy; 2 Ph.D. Program in Cellular and Molecular Biology, Department of Biology, University of Rome Tor Vergata, 00133 Rome, Italy; 3 Department of Ecological and Biological Sciences, University of Tuscia, 01100 Viterbo, Italy; 4 Division of Biological Sciences, University of Montana, Missoula, MT 59812, USA; 5 Aerospace Microbiology Research Group, Radiation Biology Department, Institute of Aerospace Medicine, German Aerospace Center (DLR), 28359 Cologne, Germany; 6 Department of Natural Sciences, University of Applied Sciences Bonn-Rhein-Sieg (BRSU), 53359 Rheinbach, Germany; 7 Mycological Section, Italian Antarctic National Museum (MNA), 16128 Genoa, Italy

**Keywords:** Antarctic ice sheet, extraterrestrial analogue, extremophiles, habitability, life detection, fungal and bacterial amplicon sequencing, planetary protection

## Abstract

The Concordia Research Station provides a unique location for preparatory activities for future human journey to Mars, to explore microbial diversity at subzero temperatures, and monitor the dissemination of human-associated microorganisms within the pristine surrounding environment. Amplicon sequencing was leveraged to investigate the microbial diversity of surface snow samples collected monthly over a two-year period, at three distances from the Station (10, 500, and 1000 m). Even when the extracted total DNA was below the detection limit, 16S rRNA gene sequencing was successfully performed on all samples, while 18S rRNA was amplified on 19 samples out of 51. No significant relationships were observed between microbial diversity and seasonality (summer or winter) or distance from the Concordia base. This suggested that if present, the anthropogenic impact should have been below the detectable limit. While harboring low microbial diversity, the surface snow samples were characterized by heterogeneous microbiomes. Ultimately, our study corroborated the use of DNA sequencing-based techniques for revealing microbial presence in remote and hostile environments, with implications for Planetary Protection during space missions and for life-detection in astrobiology relevant targets.

## 1. Introduction

Extraterrestrial analogue sites on Earth are widely exploited to support planetary exploration by testing instruments and protocols for sampling and analysis procedures for robotic explorations as well as to train astronauts for future missions on Mars [1,2]. The French–Italian Concordia Research Station located on a plateau 3233 m above sea level is a confined, isolated habitat that provides a unique location for preparatory activities for future human journey to Mars, but also to explore microbial diversity in surface snow. With its permanent inhabitation, the Concordia Station is an ideal test site to monitor the dissemination of human-associated microorganisms within the natural (pristine) surrounding habitat and its indigenous microbial diversity. Unraveling the permanence of terrestrial microorganisms in near-surface frozen environments is also of importance to protect target planets from forward contamination by human and/or robotic exploration [3,4]. Indeed, planetary protection is a main concern for space agencies and governs for a responsible exploration of other worlds [5]. Conversely, investigating the limit of microbial life detectability in frozen habitats is relevant for future exploration missions of icy bodies in the Solar System such as Europa and Enceladus [6]. Currently, life-detection technologies are based on miniaturized mass spectrometry and Raman spectroscopy [7], although, other promising techniques are under development based on nanopore sequencing for detecting nucleic acid-based life [8], and an antibody-based technique [9].

A fundamental challenge to the investigation of microbial diversity in glaciers, namely snow, glacier surface, glacier ice, and basal ice, is given by cell abundancy that increases from snow to basal ice (for a review see, [10]). A bacterial content as low as 0.2–5 × 10^3^ cells/mL was detected in the snowmelt of surface snow from the South Pole [11], while over 6 × 10^7^ cells/mL occurred in the basal ice core from Greenland [12]. The lack of cultivation technologies that mimic natural conditions is the major obstacle to culturomics and, so far, only a few hundred bacterial strains have been isolated from glacial ice from Antarctica, Greenland, Canada, Tibet, and Alaska (for a review see, [10]). For instance, more bacteria were recovered from Late Holocene ice from the Taylor Dome region in Antarctica than from ice from the Antarctic peninsula or from Greenland [12,13], while viable microorganisms were identified after a gradual melting of Vostok ice samples [14,15].

The low biomass in glacier ice samples also impairs molecular approaches to unravel microbial diversity. A few studies based on rRNA amplicon sequencing were performed, like the one on Lake Vostok accretion ice [12,16], although recently, metagenome-assembled genomes were reported for Tibetan glaciers [17] and high-throughput sequencing was used for subglacial ice samples from Svalbard [18].

The present study was performed in the framework of the BacFinder project (European Space Agency, ESA AO-13-Concordia) with the aim to unravel the environmental- and human-associated microbial diversity in the surroundings of the Concordia Station. This is the first intensive and extensive surface snow sampling performed monthly over a two-year period at three distances (10, 500, and 1000 m) from the Concordia Station, and investigated by a high-throughput sequencing approach. Emphasis was laid on the relation between microbial presence and both seasonality and distance from the Base. The challenges of monthly sampling of Antarctic snow with low microbial biomass over a 2-year period generated certain study constraints; however, this study provides a more comprehensive view of microbial communities in surface snow of the Antarctic Polar Plateau. Our intensive study corroborated the use of DNA sequencing for revealing microbial presence in remote and hostile environments, with implications for Planetary Protections and for life-detection in astrobiology relevant targets.

## 2. Materials and Methods

### 2.1. Study Area

**Figure 1:**
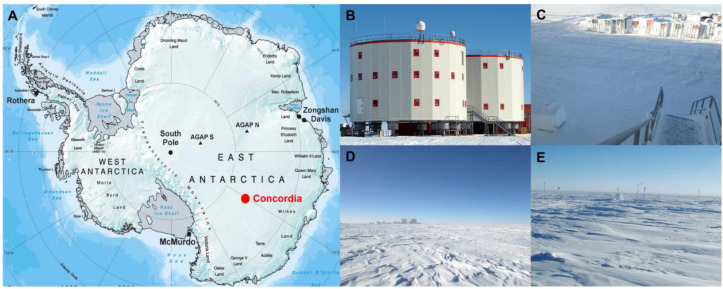
Concordia Research Station and sampling sites. (**A**) Map of Antarctica showing the Concordia Research Station at Dome C (**B**); sampling site L1 at 10 m (**C**); sampling site L2 at 500 m (**D**); and sampling site L3 at 1000 m (**E**). (**A**–**E**) photo credit: European Space Agency.

Ice samples were collected in the sampling area located at the Concordia Research Station (75°05′59″ S 123°19′56″ E) (Figure 1), which opened in 2005 as a French–Italian research facility that was built in a location called Dome C on the Antarctic Plateau, 3233 m above sea level. There, temperatures can drop to −80 °C in the winter, with a yearly average temperature of −50°C. Dome C does not experience the katabatic winds typical for the coastal regions of Antarctica because of its elevated location and its relative distance from the edges of the Antarctic Plateau. Typical wind speed in winter is 2.8 m/s. The nearest human beings are stationed some 600 km away at the Russian Vostok base, making Concordia more remote than the International Space Station. Concordia Base hosts up to 80 people during the Austral summer, including technicians, logistics, and researchers. During winter, a small group of 16 persons, named “Winter Over”, remain confined for at least 9 months.

During January–December 2015 and January–December 2016, two sampling campaigns were conducted monthly in the proximity of the Concordia station (10 m, L1), in the outer region of the research area (500 m, L2), and in the relative pristine-like area (in relative distance) to the Concordia area (1000 m, L3) as shown in Figure 1. Summer samples were collected in the period September–April; Winter samples were collected in the period May–August (see Supplementary Table S1 for more details on sampling). For each sampling site, ten replicates of snow in 50 mL-Falcons were sampled using clean, disposable nitrile gloves. Samples were preserved at −80 °C immediately upon collection and finally transported and stored at −80 °C at the German Aerospace Center (DLR, Cologne), until processing. During the processing, a total of 60 samples were yielded by pulling together 10 replicates for each site that yielded a total of 500 mL of snow for each sampling site.

### 2.2. Total DNA Extraction and Amplicon Sequencing

A total of 51 samples were processed for amplicon sequencing, while 9 (out of 60) samples were stored for culturomics experiments. Replicates were pooled resulting in a final volume of 500 mL for each sample. All samples were then melted in sterile conditions at 4 °C and filtered using Millipore filters (0.20 mm pore size). Total DNA was isolated from filters using the MOBIO Power Soil DNA Extraction kit (MOBIO Laboratories, Carlsbad, CA, USA) following the manufacturer’s instructions, and finally eluted in 50 µL. To account for possible contamination during the filtering and extraction steps, PCR-grade H_2_O was filtered through a Millipore filter and extracted following the same procedure as the snow samples.

The V4 region of the 16S rRNA and the 18S rRNA were targeted, respectively. The V4 region was amplified using the new developed barcoded F515 (GTGCCAGCMGCCGCGGTAA)/R806 (GGACTACHVGGGTWTCTAAT) primers as described by [19]; Euk_1391f (GTACACACCGCCCGTC) and EukBr (TGATCCTTCTGCAGGTTCACCTAC) primers were used to amplify the 18S rRNA region.

PCR was carried out with a total volume of 25 μL, containing 1 μL of each primer, 12.5 μL of BioMix (BioLine GmbH, Luckenwalde, Germany), 9.5 μL of nuclease-free water (Sigma–Aldrich, Gillingham, UK), and 5 ng of DNA template using an automated thermal cycler (Bio-Rad, Hercules, CA, USA). A major concern for low microbial biomass eDNA samples is the presence of contamination [20]. In addition to PCR controls, we performed PCR on extraction controls to identify any contaminants from the extraction kit. For bacteria, amplification for the V4 region followed a protocol of an initial denaturation at 94 °C for 3 min, 35 cycles of denaturation at 94 °C for 45 s, annealing at 50 °C for 1 min, extension at 72 °C for 90 s, followed by a final extension at 72 °C for 10 min. For eukaryotes, the 18S rRNA regions were amplified following initial denaturation at 94 °C for 1 min, 35 cycles of denaturation at 94 °C for 30 s, annealing at 52 °C for 30 s, extension at 68 °C for 90 s, followed by a final extension at 68 °C for 7 min. Amplicons, purified and quantified by Qubit dsDNA HS Assay Kit (Life Technologies, Carlsbad, CA, USA), were then barcoded and pooled, including the extraction and 16S rRNA amplification of PCR controls. Paired-end sequencing (2 × 300 bp) was carried out on the Illumina MiSeq platform at the Vincent J. Coates Genomics Sequencing Laboratory at the University of California (Berkeley, CA, USA), at the Edmund Mach Foundation (Trento, Italy), and at the Medical University of Graz, Center for Microbial Research (Graz, Austria).

### 2.3. Bioinformatics

The 18S rRNA and 16S rRNA amplicon sequencing datasets were processed with AMPtk: Amplicon ToolKit for Next Generation Sequence data (formally UFITS) (https://github.com/nextgenusfs/amptk accessed on 10 May 2022) v.1.0.0 [21], removing barcodes and primers from raw data. Briefly, reads were subjected to quality trimming to a maximum of 250 bp, discarding reads less than 100 bp in length, and chimera removal was performed utilizing USEARCH with default parameters v. 9.1.13 [22]. Sequence quality filtering was performed with the expected error parameter of 0.9 [23] and the cleaned dataset was clustered with UPARSE using a 97% percent identity parameter to generate the Operational Taxonomic Units (OTUs). Global singletons and rare taxa (<5 reads) were eliminated as likely false positives due to sequencing errors as described [24]. Finally, taxonomic identification was performed with hybrid database SINTAX/UTAX [22] as implemented in AMPtk. Negative controls (*n* = 3, average 718 reads) had substantially fewer reads than snow samples and these reads were taxonomically assigned to a common contaminant in extraction kits *Ralstonia*. Contaminating reads were removed from the 16S rRNA dataset before downstream analyses. Raw sequencing data have been archived in the NCBI SRA database linked to BioProject accession number PRJNA634088.

### 2.4. Downstream Analysis

Prior to further analysis, read counts were normalized; the number of reads in each sample using median sequencing depth (57,719 for 18S rRNA and 65,897 for 16S rRNA) and analyses were conducted on the rarefied dataset. The OTUs were further investigated by displaying bar plots of the relative abundance.

Statistical analyses were performed in R 2.15.1 (The R Foundation for Statistical Computing, Vienna, Austria) environment using the package phyloseq v.1.24.2 [25]. Several biodiversity indices such as richness in species, Shannon’s diversity [26], and Simpson’s dominance [27] were calculated. A pairwise comparison using the Wilcoxon rank sum test was performed to assess whether biodiversity indices differed (*p* < 0.05) among samples according to season sampling and distance from the research station.

Changes in alpha diversity and taxonomic composition were compared using one-way ANOVA and pairwise multiple comparison (Tukey test) using PAST v.2.17 software (Paleontological Statistics) [28]. A small probability *p*-value (<0.05) indicated a significant difference of community composition among all samples.

The effect of season sampling and distance was also tested using the permutational ANOVA (PERMANOVA) with 999 numbers of permutations applied with the “adonis” function in the “vegan” v.2.5-4 [29] package. Changes in community composition were displayed with PCoA ordination plots using the unweighted UniFrac distance.

Venn diagrams were constructed to show the number of 16S rRNA and 18S rRNA shared and unique OTUs, based on OTU tables normalized, using the VENNY tool [30].

## 3. Results

### 3.1. Raw Data Processing

DNA concentration was below the detection limit (0.005 ng/μL) for most of the samples. The 16S rRNA gene amplicon sequencing from 51 samples produced a total of 6,257,600 paired-end reads. Clustering of Operational Taxonomic Units (OTUs) was performed at 97% identity threshold, resulting in 5,187,083 reads mapped to OTUs. Sequences were grouped into 7171 OTUs (Supplementary Table S2). After singletons and rare taxa removal (9 out of 4536 OTUs), and normalization using median sequencing depth, a total of 2635 quality-filtered bacterial OTUs were obtained.

For eukaryotes, the 18S rRNA sequence reads were obtained from 19 samples (out of 54) and resulted in 1,305,012 sequences and upon filtering, 1,281,800 reads were mapped into 105 quality-filtered OTUs (Supplementary Table S3). The OTUs table was then normalized among samples using median sequencing depth.

In the 16S rRNA dataset, the rarefaction curve of total OTUs per sample reached a plateau only for a few samples, while the rarefaction curves generated from 18S rRNA datasets never reached a plateau (Supplementary Figure S1).

### 3.2. Taxonomy Structure and Composition

**Figure 2:**
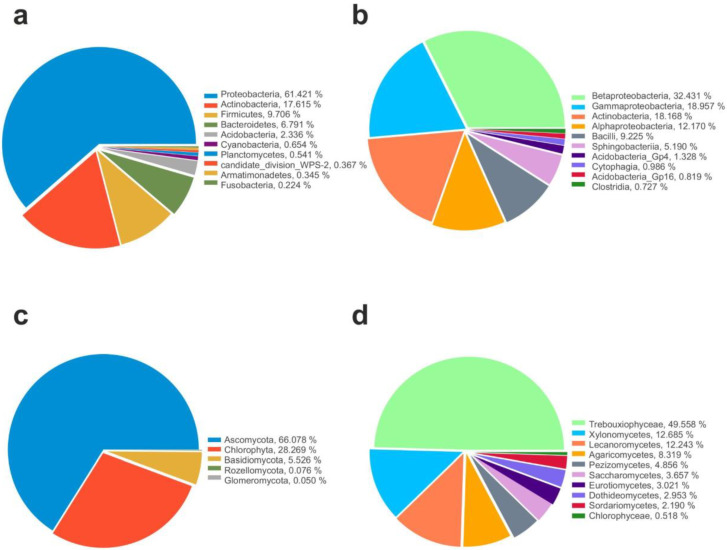
Microbial community composition. Relative abundance of top 10 identified Phyla and Classes in 16S (**a**,**b**) and 18S (**c**,**d**) datasets.

The majority of the identified 16S sequences recovered among all samples belonged to Proteobacteria (57%), followed by Cyanobacteria (15%), Actinobacteria (11%), Firmicutes (9%), Bacteroides (4%), and Acidobacteria (1%) as shown in Figure 2a. Members belonging to Planctomycetes, Verrucomicrobia, Gemmatimonadetes, Nitrospirae, Deinococcus-Thermus, Chloroflexi, Armatimonadetes, Fusobacteria, Parcubacteria, candidate division WPS-1, Chlamydiae, BRC1, Microgenomates, Spirochaetes, and Diapherotrites have been retrieved in the lowest percentage (<1%). Additionally, several archaeal taxa such as members belonging to Woesearchaeota, Thaumarchaeota, Pacearchaeota, and Euryarchaeota were also found, even if at lowest abundance (<1%).

As shown in Figure 2b, among the classes, Betaproteobacteria (30%) was the most abundant, followed by Gammaproteobacteria (18%), Actinobacteria (11%), Alphaproteobacteria (9%), Bacilli (9%), and Sphingobacteriia (2%). Members belonging to other classes were less frequent across the dataset (<1%), as follows: Acidobacteria (0.80%), Clostridia (0.69%), Flavobacteriia (0.61%), Acidobacteria Gp16 (0.50%), Cytophagia (0.41%), Cyanobacteria (0.40%), Planctomycetia (0.35%), Armatimonadia (0.26%), Fusobacteria (0.22%), Bacteroidia (0.19%), Gemmatimonadetes (0.14%), Acidobacteria Gp6 (0.13%), Spartobacteria (0.11%), and Deinococci (0.10%).

Instead, among eukaryotes, most of the identified sequences belonged to fungi, in particular the phylum Ascomycota that represents the most abundant phylum (66%) for fungi and occurred in all analyzed samples, followed by Basidiomycota (5%), while members belonging to Glomeromycota and Rozellomyconta were present in the lowest percentage (<1%). Algae belonging to Chlorophyta were represented by 28% (Figure 2c).

Among the fungal classes, the most representative were Lecanoromycetes (12.2%), Xylonomycetes (12.7%), Agaricomycetes (8.37%), Pezizomycetes (4.9%), Saccharomycetes (3.7%), Eurotiomycetes (3.1%), Dothideomycetes (2.9%), and Sordariomycetes (2.2%), while algae were represented by Trebouxiophyceae (49.5%) and Chlorophyceae (0.6%), respectively (Figure 2d).

We further examined the distribution of both 16S rRNA and 18S rRNA taxa, showing that their relative abundance, in general, was highly variable across all samples; indeed, most taxa were present intermittently among sampled sites (Supplementary Figures S2 and S3).

**Figure 3:**
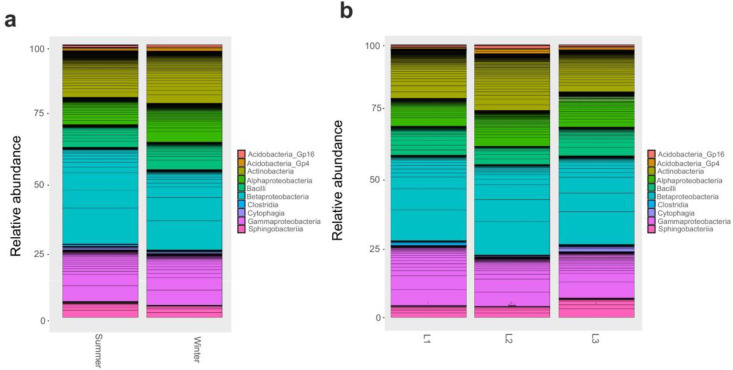
Barplot of Class relative abundances in 16S rRNA dataset. Barplot based on the sampling season as shown in supplementary Table S1: Summer and Winter (**a**). Barplot based on sampling distances from Concordia Research Station: L1 = 10 m, L2 = 500 m, and L3 = 1.000 m (**b**).

As OTUS were unidentified at a finer level, we proceeded with a comparison of taxonomic composition by distance and seasonality at phylum and class level. We found that changes in bacterial community composition at phylum level was not observed. Instead, at class level, Gammaproteobacteria and Alphaproteobacteria significantly (*p* < 0.05) varied according to season; in particular, Gammaproteobacteria were most abundant during winter, while the latter was in the summer (Figure 3a); moreover, Gammaproteobacteria were enriched (*p* < 0.05) in the proximity of the research station (L1, 10 m) (Figure 3b).

**Figure 4:**
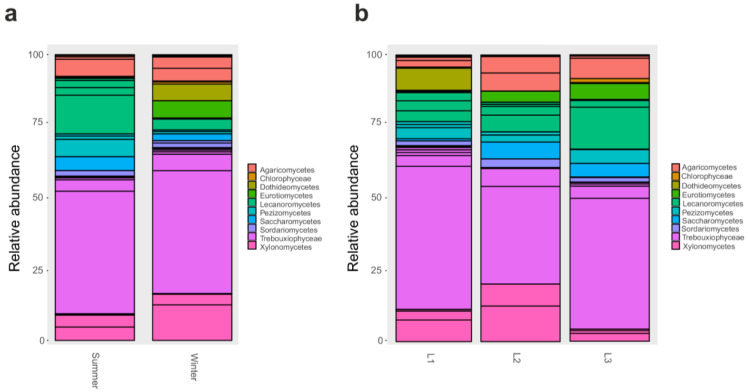
Barplot of Classes relative abundances in 18S rRNA dataset. Barplot based on the sampling season, as shown in Table S1: Summer and Winter (**a**). Barplot based on sampling distances from Concordia Research Station: L1 = 10 m, L2 = 500 m, and L3 = 1.000 m (**b**).

When 18S rRNA dataset was examined, we found that several classes significantly varied (*p* < 0.05) according to the season: in particular, Eurotiomycetes were particularly abundant in the winter as well as Xylonomycetes, Agaricomycetes, Chlorophyceae, and Dothideomycetes (Figure 4a). Instead, when comparing samples collected at different distances from the research station, we reported that Xylonomycetes were particularly abundant in L2 and L3, contrary to Lecanoromycetes that decreased in those two groups. In contrast, Dothideomycetes was predominant in L2, while Chlorophyceae were present mainly in L1 (*p* < 0.05) (Figure 4b).

### 3.3. Preliminary Data of Anthropogenic Effect on Alpha and Beta Diversity

**Figure 5:**
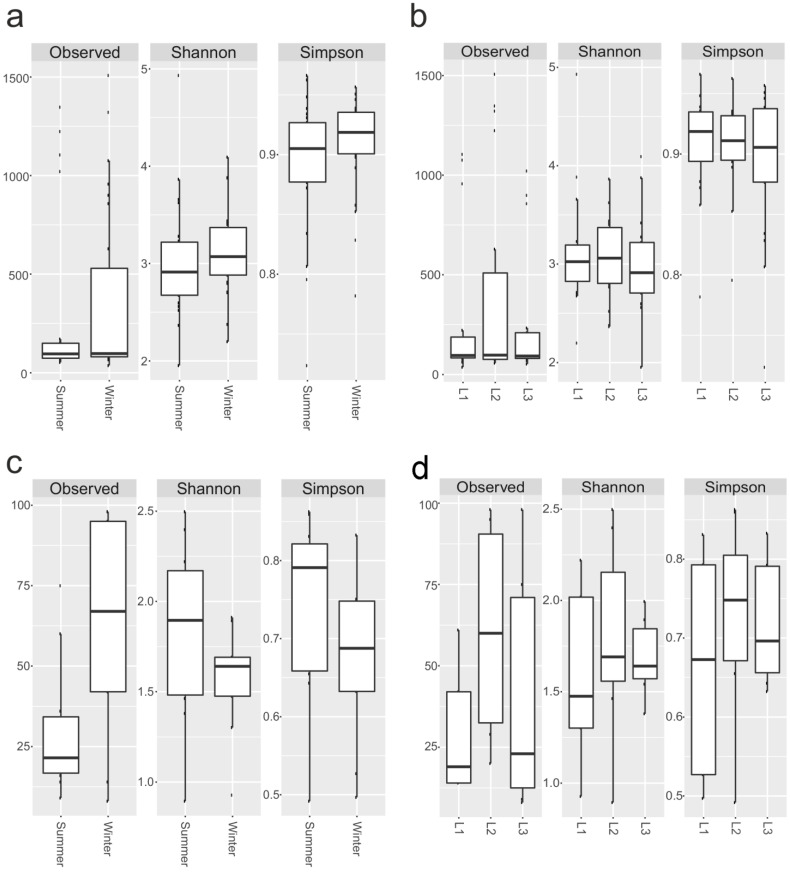
Biodiversity indices. Observed OTUs, Shannon’s and Simpson’s indices for 16S, based on season (**a**), distances (**b**), and 18rRNA datasets (**c**,**d**). Boxplots show 25th and 75th percentile, while error bars 1st and 99th percentile.

The number of OTUs and biodiversity indices such as Shannon’s and Simpson’s metrics were calculated for each sample, as well as for 16S rRNA and 18S rRNA components. For prokaryotes, the number of observed OTUs ranged from 37 (sample S6) to 1507 (S12), while biodiversity varied from 1.95 (S18) to 4.93 (S16) and from 0.72 (S8) to 0.96 (S16 and S1) for Shannon’s and Simpson’s indices, respectively (Figure 5a,b; Supplementary Table S4).

Overall, the 18S rRNA biodiversity was lower than the 16S rRNA counterpart (Figure 5a,b). The richness ranged from 8 (sample 18S_S9) to 98 (18S_S12 and 18S_S14), Shannon’s index from 0.89 (18S_S17) to 2.49 (18S_S2), and Simpson’s index from 0.49 (18S_S17) to 0.86 (18S_S2) (Figure 5c,d; Supplementary Table S5).

We did not observe any significant change (Wilcoxon-Mann-Whitney) in 16S rRNA and 18S rRNA biodiversity according to site and sampling.

**Figure 6:**
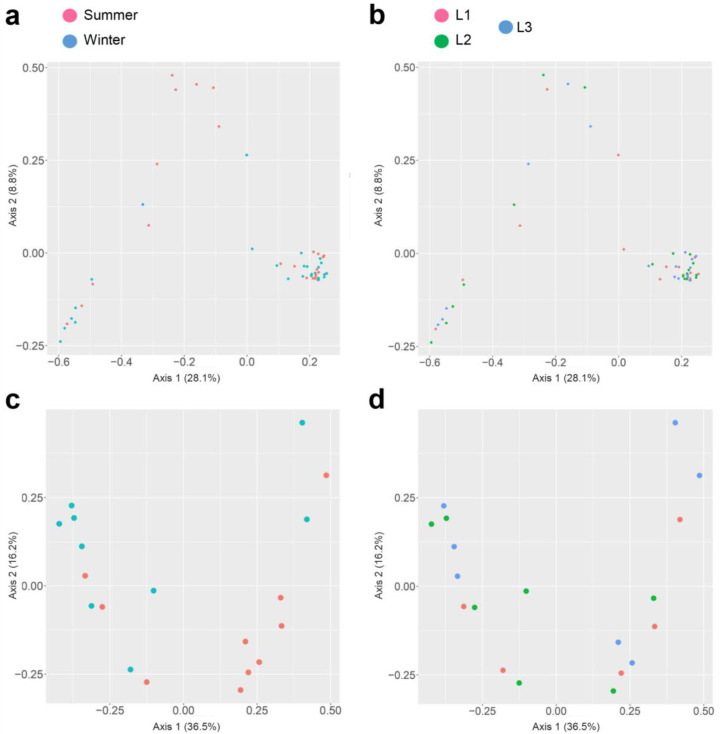
Principal Coordinate Analysis (PCoA) plot of the beta diversity distances obtained from the unweighted UNIFRAC analysis of Observation, Shannon index, and Simpson index. PCoA of 16S rRNA sequences based on season (**a**), distances (**b**), and PCoA of 18S rRNA sequences based on season (**c**) and distances (**d**).

In both 16S rRNA and 18S rRNA data sets, the beta diversity was assessed from unweighted UniFrac distance and presented with PCoA plots and PERMANOVA statistical analysis. The effect of season sampling and distance were not found to be significant in 16S rRNA (*p*-value > 0.05), while the 18S rRNA samples were found to be significant (*p*-value = 0.028 < 0.05) for seasons only (Figure 6).

### 3.4. Unique and Shared Taxa According to Distances and Season

**Figure 7:**
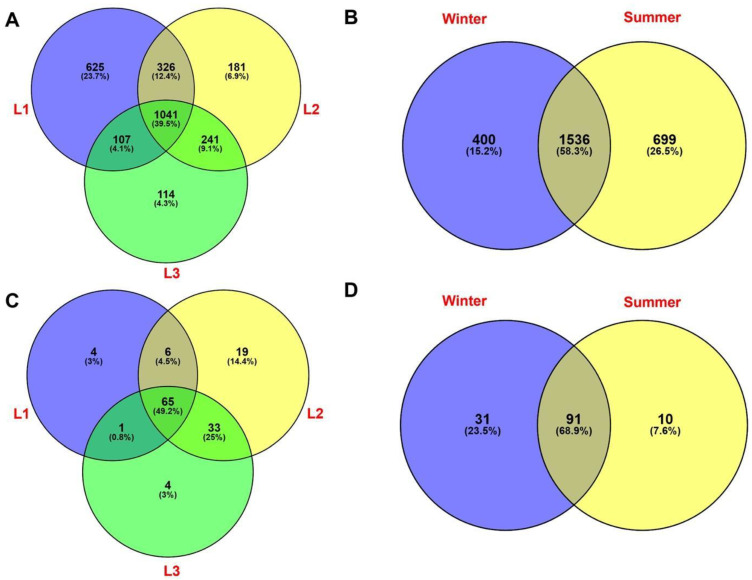
Venn diagram showing the distribution of bacterial taxa (16S rRNA dataset) relative to distances (**A**) and season (**B**) data. Venn diagrams showing the distribution of eukaryotic taxa (18S rRNA taxa dataset) on distances and season data, respectively (**C**,**D**). Percentages of both shared and unique OTUs are shown in parentheses.

Venn diagrams were used to explore the percentage of shared and unique identified taxa at class level among all samples. As shown in Figure 7A, a substantial fraction of prokaryotic OTUs (1041 OTUs, 39.5%) were shared among the three sampling distances s, while 625 (23.7%), 181 (6.9%), and 114 (4.3%) OTUs were unique for L1, L2, and L3, respectively. When seasonality is considered, we found that almost 60% of prokaryotic taxa (1536 OTUs) were shared between winter and summer; further, a lower percentage of unique prokaryotes was found in the two seasons (400 OTUs, 15.2% in winter and 699 OTUs, 26.5% in summer, respectively) (Figure 7B).

For eukaryotes, Venn diagram showed that almost half (49.2%) of the total OTUs were shared among the three distances, while only 4 taxa (3%) were unique to L1 and L3 distances, including members of Sordariomycetes (*Trichoderma* sp.) and Chlorophyta, while 19 (14.9%) taxa were found exclusively in L2 (e.g., members belonging to Chytridiomycota, Pezizales, *Aspergillus* sp., and Trebouxiophyceae) (Figure 7C). Figure 7D showed that a few taxa (10 OTUs, 7.6%) were found in summer, exclusively; while a greater percentage of taxa (31 OTUs, 23.5%) were unique for the winter. The highest fraction of OTUs (91, 68.9%) was shared between the two seasons.

## 4. Discussion

Glacial habitats are among the most challenging natural environments for life. In particular, the ice sheet of the Antarctic Polar Plateau, the ancient, most isolated, stable and coldest icy environment on Earth, represents a peculiar case where liquid water essential for active life is virtually absent. This work contributes to deepening our knowledge on prokaryotic (V4 region of 16S rRNA gene) and eukaryotic (V4 region of 18S rRNA gene) microbial assemblages inhabiting the Antarctic ice shelf of the Polar Plateau, accounted as the most remote base on Earth, during a 2-year monthly sampling and over a distance of 1 km from the Concordia Research Station. The only microbiological study to date that is available on this habitat was performed several years ago and was based on a single snow sampling at 2 km distance from the Concordia Research Station and investigated only the bacterial 16S rRNA component. In that study, it was reported that the snow melt samples may contain approximately 3.4 × 10^2^ cells mL^−1^ of autotrophic and heterotrophic bacteria and a negligible amount of eukaryotes [31].

In the present study, the PCR amplification and subsequent high throughput sequencing was performed on 51 samples for the 16S rRNA target, while only 19 samples were successfully targeted for the 18S rRNA amplification. Indeed, despite organisms occurring in an environment at a very low abundance, in the present study, microbial diversity could be revealed by PCR for Illumina sequencing of the almost undetectable (0.005 ng/µL) amounts of DNA that were obtained from each sampling site (500 mL of surface snow/site).

Among prokaryotes, members of phylum Proteobacteria resulted largely as the most abundant followed by Actinobacteria and Firmicutes. The first two, in particular, are reported among the most abundant prokaryotic representatives both in Earth’s major habitat types [32] and airborne bacteria in atmospheric bioaerosols [33,34]. Proteobacteria were also found to be dominant by Michaud and collaborators (2014) in a snow sample near Concordia Station and in other extreme cold environments [35,36,37,38,39]. Conversely, Cyanobacteria, despite being dominant in Antarctic terrestrial environments and having the capability to directly influence components of the cryosphere [40], were found only in a single snow sample (S7), at a 500-m distance from the Concordia Station. This very low incidence of autotrophic prokaryotes does not support the hypothesis of their role as primary producers and ecosystem engineers in an active microbial community in the Antarctic snow of the Polar Plateau as previously suggested [31].

Eukaryotes were found at very low abundance and were mostly represented by fungi. This is not surprising because most of these microorganisms produce abundant airborne propagules that are easily transported, even throughout remote environments. The majority belonged to the most abundant fungal phylum Ascomycota, which is by far the largest of the fungal Kingdom, hosting numerous abundantly sporulating fungi. Among the most abundant fungal classes found, Eurotiomycetes, whose members are of the *Aspergillus*/*Penicillium* group, are known to be globally distributed and copiously producing airborne propagules. The class Dothideomycetes is the widest in Ascomycota producing bitunicate asci; frequently encountered taxa in this class, such as *Aureobasidium*, *Cladosporium*, or *Hortaea* have common characteristics of adaptation or resistance to low temperature and high osmotic pressure, which may be essential to survival under the icy conditions of the Polar Plateau. Other common taxa in this class were, instead, typically plant associated as for the genus *Phoma*.

Surprisingly, we found members of the class Xylonomycetes, only relatively recently discovered and described, which are exclusively found associated as endophytes with plants of remote forests in Peru [41]. Despite the fact that they also are sporulating and producing picnidia even under lab conditions, they are not widespread fungi and their random presence in ice samples of the Polar Plateau is difficult to explain. Their presence may be more probably ascribed to their capacity to spread globally since it is becoming evident that much of the microbial life within remote environments, including Antarctica, is transported by air currents [42]. This is true even for Chlorophyceae algae, here found in a relatively high abundance, which are reported to be easily transported overseas among continents [43].

Our interpretation of the coincidental occurrence of microbial eukaryotes found was also corroborated by the almost undetectable presence and lack of any correlation with distance to the Concordia Station. Even their apparently higher presence in the winter season, when the anthropogenic impact is surely much lower, is difficult to explain, if not as an accidental datum due to the high variability among samples and the very insubstantial presence of DNA.

OTUs richness and other biodiversity indices such as Shannon’s and Simpson’s metrics were calculated for each sample for comparative purposes to test whether different conditions (i.e., distance from the station and seasonality) significantly impact community composition. Overall, this work suggested that while harboring low microbial diversity, the investigated snow samples were characterized by heterogeneous microbiomes. Indeed, a considerable amount of heterogeneity in alpha-diversity has been observed between samples, both for prokaryotes and eukaryotes. Specifically, in our survey, richness and Shannon’s index values showed relatively low diversity; they significantly varied among all samples: number of observed species ranged from 37 to 1507 and 8 to 98 in fungi and bacteria, respectively, while Shannon’s diversity values ranged from 1.95 to 4.93 and 0.89 to 2.49 in the two assemblages, respectively. However, this high variability was correlated to neither distance from the station nor sampling seasons (*p* > 0.05). Interestingly, snow samples were found to have a core (i.e., OTUs present in at least 75% of the samples) microbiome which persisted regardless of the environmental factors and level of human activity.

Taken together, this preliminary study provided a high-throughput sequencing of the prokaryotic and eukaryotic icy communities occurring in the Antarctic plateau in the vicinity of the Concordia Research Station. In order to provide new insights into the anthropogenic impact on these fragile ecosystems, the taxonomy and community composition of the surface snow samples were evaluated by considering the distance from the Concordia Research Station and the seasonality of the sampling.

A further and more extensive sampling is necessary to better investigate the microbial diversity in superficial snow over a prolonged period of time. Therefore, in order to increase the biomass to obtain adequate amounts of DNA to enable a comprehensive meta-community profiling, the crew members of the Concordia Research Station collected during the 2018–2019 campaign 15 samples of 50 mL-Falcon, instead of 10 samples of 50 mL-Falcon as in the 2015–2016 campaign.

Data suggested that if present, the anthropogenic impact should have been below the detectable limit. Snow samples had variable microbial assemblages that did not associate with changes in seasonality or distance from station, suggesting a transient existence for resident microbes that might not be significantly influenced by environmental variables. Furthermore, RNA analysis associated with a metagenomic approach could also provide insights into the metabolic status of the microbial communities.

Finally, our study corroborated the use of DNA sequencing-based techniques for revealing microbial presence in remote and hostile environments, with implications for Planetary Protection during space missions and for the development of life–life-detection instrumentations for astrobiological relevant targets such as Mars and the icy moons Europa and Enceladus.

## Data Availability

Data available in a publicly accessible repository. NCBI SRA database linked to BioProject accession number PRJNA634088.

## Supplementary Materials

The following supporting information can be downloaded at: https://www.mdpi.com/article/10.3390/life13010113/s1, Figure S1: Rarefaction curve of total OTUs, 16S rRNA (A) and 18S rRNA (B) datasets; Figure S2: Barplot of Class in 16S rRNA dataset, Barplot based on season, as shown in Supplementary Table S1: Summer and Winter (A), Barplot based on distance (L1 = 10 m, L2 = 500 m and L3 = 1000 m) from Concordia Research Station (B); Figure S3: Barplot of Class 18S rRNA dataset. Barplot based on season, as shown in Supplementary Table S1: Summer and Winter (A), Barplot based on distance (L1 = 10 m, L2 = 500 m and L3 = 1000 m) from Concordia Research Station (B); Table S1: Distances at Concordia Station and metadata. Data include samples correspondence, distance from research station, coordinates, season and sampling date are reported. The samples for which 16S rRNA and 18S rRNA amplicon sequencing was successful are reported in green, while the unsuccessfully ones are in in red; Table S2: 16S rRNA taxonomy. Taxonomy assignment (results at 97% of identity) was performed with SINTAX/UTAX databases (Edgar, 2010); Table S3: 18S rRNA taxonomy. Taxonomy assignment (results at 97% of identity) was performed with SINTAX/UTAX databases (Edgar, 2010); Table S4: 16S rRNA biodiversity. Observed OTUs Shannon’s and Simpson’s indices for each sample; Table S5: 18S rRNA biodiversity. Observed OTUs Shannon’s and Simpson’s indices for each sample.
