# Supplementary material for: Snow Surface Microbial Diversity at the Detection Limit within the Vicinity of the Concordia Station, Antarctica
Source: Life (Basel). 2022 Dec 30;13(1):113. doi: 10.3390/life13010113 (PMC9863605; doi:10.3390/life13010113)
Supplement: Supplementary file 1 [file life-13-00113-s001.zip › Supplementary Figures.pdf]

Supplementary Figure legends

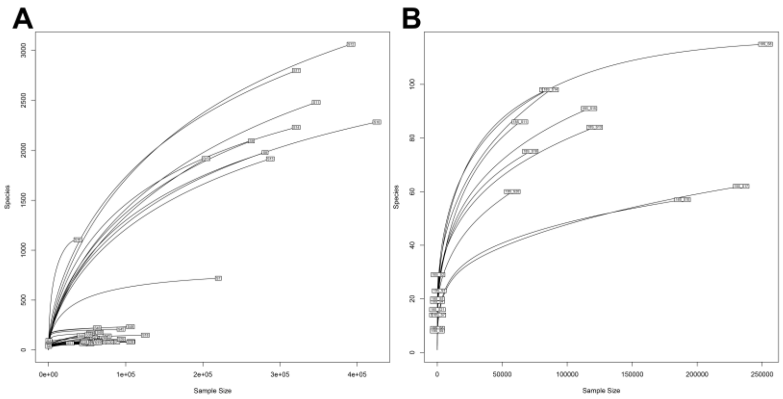

Figure S1. Rarefaction curve of total OTUs. 16S rRNA (**A**) and 18S rRNA (**B**) datasets.

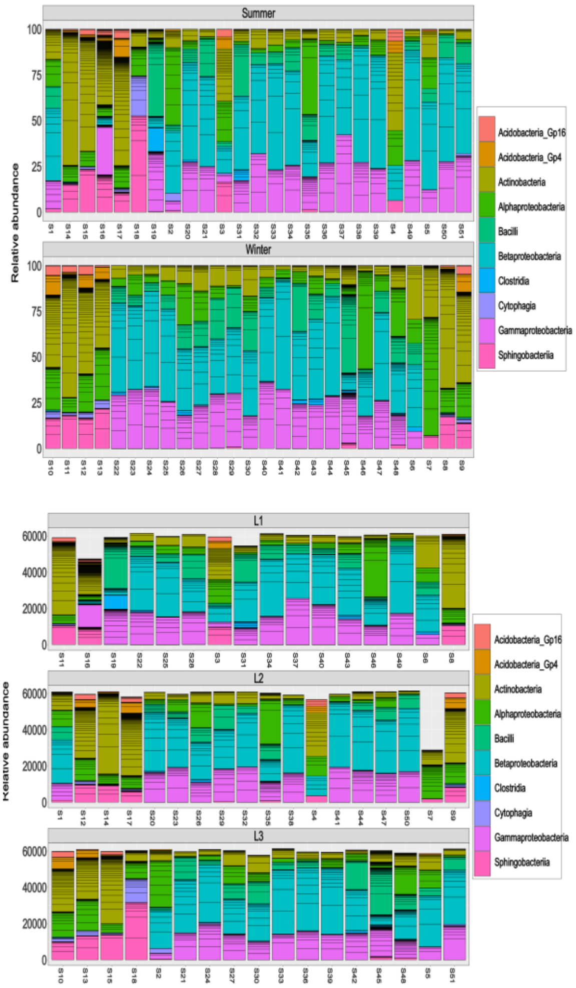

Figure S2. Barplot of Class in 16S rRNA dataset. Barplot based on season, as shown in supplementary table S1: Summer and Winter (A). Barplot based on distance (L1=10 m, L2=500 m and L3=1,000 m) from Concordia Research Station (B).

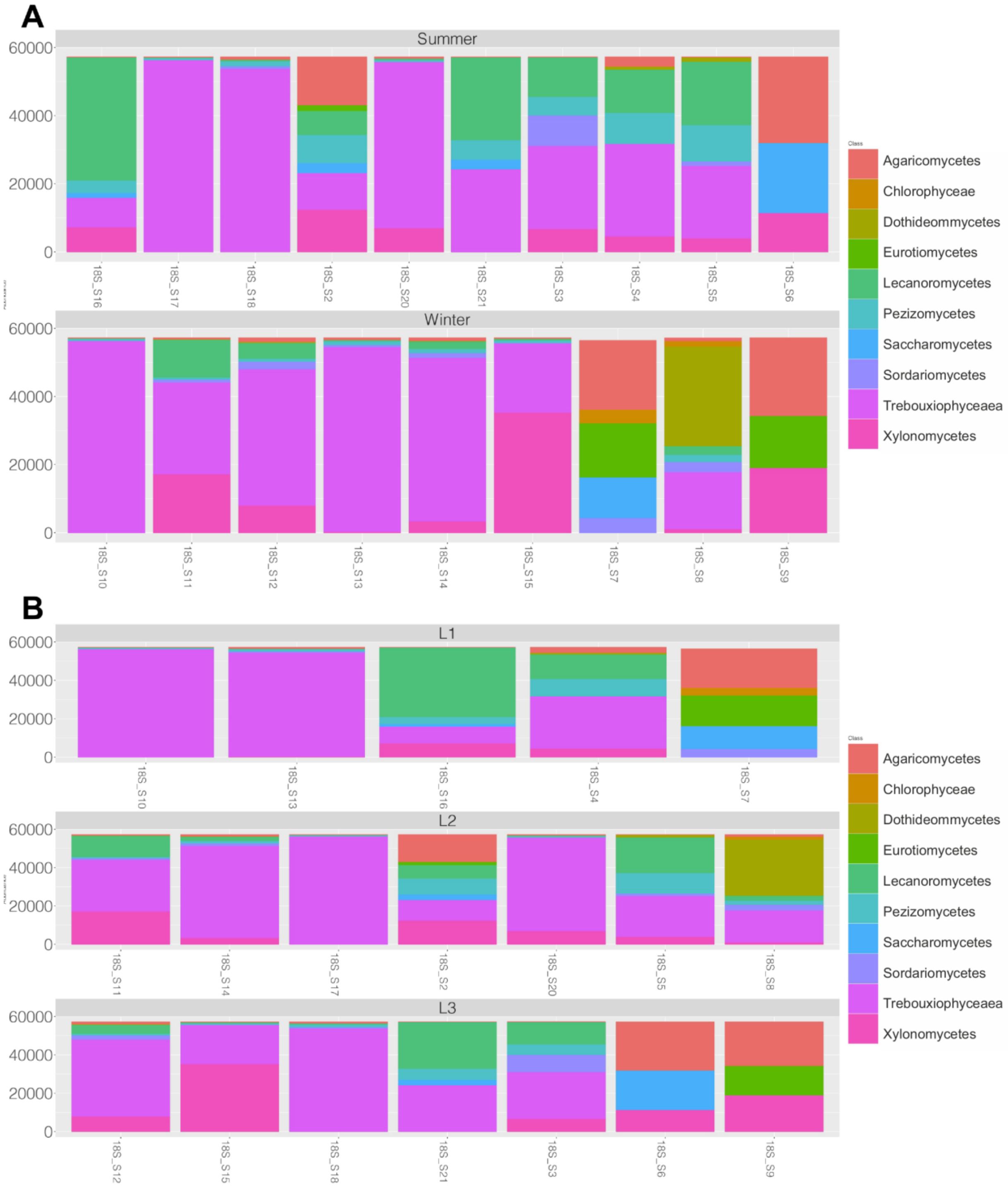

Figure S3. Barplot of Class 18S rRNA dataset. Barplot based on season, as shown in supplementary table S1: Summer and Winter (A). Barplot based on distance (L1=10 m, L2=500 m and L3=1,000 m) from Concordia Research Station (B).
