# Supplementary material for: Snow Surface Microbial Diversity at the Detection Limit within the Vicinity of the Concordia Station, Antarctica
Source: Life (Basel). 2022 Dec 30;13(1):113. doi: 10.3390/life13010113 (PMC9863605; doi:10.3390/life13010113)
Supplement: Supplementary file 1 [file life-13-00113-s001.zip › Table S1.pdf]

Table S1. Distances at Concordia Station and metadata. Data include samples correspondence, distance from research station, coordinates, season and sampling date are reported. The samples for which 16S rRNA and 18S rRNA amplicon sequencing was successful are reported in green, while the unsuccessfully ones are in in red.

16S Sample correspondence	18S Sample correspondence	Distance from Concordia (m)	Distance L1-L3	Sampling season	Sampling date	Coordinates
16S_S1	18S_S1	500	L2	Winter	Oct'15	75.09996S 123.33322E
16S_S2	18S_S2	1000	L3	Winter	Oct'15	75.10521S 123.31152E
16S_S3	18S_S3	10	L1	Winter	Feb'15	75.09996S 123.33322E
16S_S4	18S_S4	500	L2	Winter	Feb'15	75.09996S 123.33322E
16S_S5	18S_S5	1000	L3	Winter	Feb'15	75.10521S 123.31152E
16S_S6	18S_S6	10	L1	Winter	April'16	75.09996S 123.33322E
16S_S7	18S_S7	500	L2	Winter	April'16	75.09996S 123.33322E
16S_S8	18S_S8	1000	L3	Winter	June'16	75.10521S 123.31152E
16S_S9	18S_S9	10	L1	Winter	June'16	75.09996S 123.33322E
16S_S10	18S_S10	500	L2	Winter	June'16	75.09996S 123.33322E
16S_S11	18S_S11	1000	L3	Winter	Aug'16	75.10521S 123.31152E
16S_S12	18S_S12	10	L1	Winter	Aug'16	75.09996S 123.33322E
16S_S13	18S_S13	500	L2	Winter	Aug'16	75.09996S 123.33322E
16S_S14	18S_S14	1000	L3	Winter	Oct'16	75.10521S 123.31152E
16S_S15	18S_S15	10	L1	Winter	Oct'16	75.09996S 123.33322E
16S_S16	18S_S16	500	L2	Summer	Dec'16	75.09996S 123.33322E
16S_S17	18S_S17	1000	L3	Summer	Dec'16	75.10521S 123.31152E
16S_S18	18S_S18	10	L1	Summer	Dec'16	75.09996S 123.33322E
16S_S19	18S_S19	500	L2	Summer	Mar'15	75.09996S 123.33322E
16S_S20	18S_S20	1000	L3	Summer	Mar'15	75.10521S 123.31152E
16S_S21	18S_S21	10	L1	Summer	Mar'15	75.09996S 123.33322E
16S_S22	18S_S22	500	L2	Winter	May'15	75.09996S 123.33322E
16S_S23	18S_S23	1000	L3	Winter	May'15	75.10521S 123.31152E
16S_S24	18S_S24	10	L1	Winter	May'15	75.09996S 123.33322E
16S_25	18S_S25	500	L2	Winter	Jul'15	75.09996S 123.33322E
16S_S26	18S_S26	1000	L3	Winter	Jul'15	75.10521S 123.31152E
16S_S27	18S_S27	10	L1	Winter	Jul'15	75.09996S 123.33322E
16S_S28	18S_S28	500	L2	Winter	Sept'15	75.09996S 123.33322E
16S_S29	18S_S29	1000	L3	Winter	Sept'15	75.10521S 123.31152E
16S_S30	18S_S30	10	L1	Winter	Sept'15	75.09996S 123.33322E
16S_S31	18S_S31	500	L2	Summer	Nov'15	75.09996S 123.33322E
16S_S32	18S_S32	1000	L3	Summer	Nov'15	75.10521S 123.31152E
16S_S33	18S_S33	10	L1	Summer	Nov'15	75.09996S 123.33322E
16S_S34	18S_S34	500	L2	Summer	Jan'16	75.09996S 123.33322E
16S_S35	18S_S35	1000	L3	Summer	Jan'16	75.10521S 123.31152E
16S_S36	18S_S36	10	L1	Summer	Jan'16	75.09996S 123.33322E
16S_S37	18S_S37	500	L2	Summer	Mar'16	75.09996S 123.33322E
16S_S38	18S_S38	1000	L3	Summer	Mar'16	75.10521S 123.31152E
16S_S39	18S_S39	10	L1	Summer	Mar'16	75.09996S 123.33322E
16S_S40	18S_S40	500	L2	Winter	May'16	75.09996S 123.33322E
16S_S41	18S_S41	1000	L3	Winter	May'16	75.10521S 123.31152E
16S_S41	18S_S42	10	L1	Winter	May'16	75.09996S 123.33322E
16S_S43	18S_S43	500	L2	Winter	Jul'16	75.09996S 123.33322E
16S_S44	18S_S44	1000	L3	Winter	Jul'16	75.10521S 123.31152E
16S_S45	18S_S45	10	L1	Winter	Jul'16	75.09996S 123.33322E

16S_S46	18S_S46	500	L2	Winter	Sept'16	75.09996S 123.33322E
16S_S47	18S_S47	1000	L3	Winter	Sept'16	75.10521S 123.31152E
16S_S48	18S_S48	10	L1	Winter	Sept'16	75.09996S 123.33322E
16S_S49	18S_S49	500	L2	Summer	Nov'16	75.09996S 123.33322E
16S_S50	18S_S50	1000	L3	Summer	Nov'16	75.10521S 123.31152E
16S_S51	18S_S51	10	L1	Summer	Nov'16	75.09996S 123.33322E
