# Supplementary material for: Snow Surface Microbial Diversity at the Detection Limit within the Vicinity of the Concordia Station, Antarctica
Source: Life (Basel). 2022 Dec 30;13(1):113. doi: 10.3390/life13010113 (PMC9863605; doi:10.3390/life13010113)
Supplement: Supplementary file 1 [file life-13-00113-s001.zip › Table S4.pdf]

Table S4. 16S rRNA biodiversity. Observed OTUs Shannon’s and Simpson’s indices for each sample.

Sample ID	Observed OTUs	Shannon's index	Simpson's index
S1	73	3.62	0.96
S2	81	2.59	0.80
S3	96	3.65	0.94
S4	57	3.27	0.93
S5	53	3.21	0.93
S6	37	3.13	0.93
S7	628	3.42	0.93
S8	956	2.19	0.78
S9	1321	3.40	0.90
S10	899	3.41	0.91
S11	1076	2.93	0.85
S12	1507	3.43	0.89
S13	857	2.70	0.82
S14	1223	2.36	0.79
S15	1020	2.71	0.83
S16	1105	4.93	0.96
S17	1347	3.86	0.93
S18	149	1.95	0.72
S19	170	3.22	0.89
S20	75	3.03	0.92
S21	81	2.98	0.92
S22	79	2.98	0.91
S23	73	3.08	0.92
S24	81	2.80	0.90
S25	65	2.70	0.90
S26	82	3.17	0.93
S27	93	3.27	0.94
S28	92	3.06	0.93
S29	80	3.00	0.93
S30	79	3.16	0.93
S31	85	3.04	0.90
S32	83	2.76	0.91
S33	74	2.55	0.87
S34	62	2.70	0.87
S35	141	3.19	0.89
S36	120	2.91	0.90
S37	127	2.86	0.92
S38	152	2.83	0.90
S39	110	3.03	0.90
S40	176	3.00	0.91
S41	102	2.37	0.85
S42	88	2.88	0.90
S43	91	3.18	0.93

S44	144	2.79	0.88
S45	209	3.87	0.95
S46	221	3.88	0.93
S47	93	2.88	0.90
S49	233	4.09	0.95
S50	96	2.67	0.87
S51	74	2.51	0.89
S50	68	2.59	0.87
