# Supplementary material for: Snow Surface Microbial Diversity at the Detection Limit within the Vicinity of the Concordia Station, Antarctica
Source: Life (Basel). 2022 Dec 30;13(1):113. doi: 10.3390/life13010113 (PMC9863605; doi:10.3390/life13010113)
Supplement: Supplementary file 1 [file life-13-00113-s001.zip › Table S5.pdf]

Table S5. 18S rRNA biodiversity. Observed OTUs Shannon's and Simpson's indices for each sample.

Sample ID	Observed OTUs	Shannon's index	Simpson's index
18S_S2	29	29	0.86
18S_S3	23	23	0.66
18S_S4	19	19	0.83
18S_S5	20	20	0.85
18S_S6	9	9	0.78
18S_S7	14	14	0.52
18S_S8	95	95	0.75
18S_S9	8	8	0.83
18S_S10	42	42	0.49
18S_S11	86	86	0.74
18S_S12	98	98	0.63
18S_S13	61	61	0.67
18S_S14	98	98	0.68
18S_S15	67	67	0.69
18S_S16	14	14	0.79
18S_S17	36	36	0.49
18S_S18	75	75	0.64
18S_S20	60	60	0.65
18S_S21	16	16	0.79
